# Home food environment and associations with weight and diet among U.S. adults: a cross-sectional study

**DOI:** 10.1186/s12889-021-11102-2

**Published:** 2021-06-01

**Authors:** Michelle C. Kegler, April Hermstad, Regine Haardörfer

**Affiliations:** Emory Prevention Research Center, Rollins School of Public Health, Emory University, 1518 Clifton Road NE, Atlanta, GA 30322 USA

**Keywords:** Home environment, Obesity, Diet quality, Food inventory, Fruit and vegetable intake, Cross-sectional survey

## Abstract

**Background:**

The home provides the physical and social context for the majority of eating behaviors for U.S. adults. This study describes eleven dimensions of the home food environment among a national sample of U.S. adults and identifies which are associated with diet quality and overweight/obesity.

**Methods:**

A national sample of U.S. adults ages 18 to 75 was recruited from an online survey panel. Respondents (*n* = 4942) reported on foods available in the home, including 1) fruit and vegetables, 2) salty snacks/sweets, 3) less healthy beverages, as well as 4) food placement, 5) shopping practices for fruits and vegetables, 6) food preparation, 7) portion control methods, 8) family meals from restaurants, 9) family household practices around TV and eating, 10) presence of a TV in the dining area, and 11) ownership of a scale. Self-reported height and weight, fruit and vegetable intake, and percent calories from fat were also assessed.

**Results:**

Mean household size was 2.6, 32.7% had children in the home, and 23.1% lived alone. The majority were White (67.7%), with 12.3% Black and 14.3% Hispanic. Mean age was 44.4 and 48.3% were men. In multivariable models, seven features of the home food environment were associated with meeting the recommended fruit and vegetable intake guidelines, with food placement, meal preparation, frequency of shopping for fruit, and a greater variety of fruits and vegetables available in the home most strongly associated. Eight of 11 features were associated with percent energy from fat, including restaurant food for family meals, salty snacks and sweets availability, less healthy beverages availability, food placement, meal preparation, frequency of shopping for fruit, family eating with the TV on, and having a TV in the dining area. More diverse fruit and vegetable availability was associated with lower odds of overweight/obesity, and more frequent family eating while watching TV was associated with increased odds of overweight/obesity.

**Conclusion:**

Targeting these dimensions of the home food environment may be a promising approach for future intervention research.

## Background

The majority of Americans do not meet the U.S. Dietary Guidelines, and identifying potential leverage points for improving diet continues as a public health priority [1]. Because a majority of food is still consumed at home, even with the proportion diminishing in the past few decades the home is a proximal environment to much of the eating behavior of Americans [2]. A number of studies have examined one or two aspects of the home environment, such as foods available in the home or frequency of shared family meals [3–9], but relatively few have examined a comprehensive set of home environment features and their possible association with weight and diet quality. Those that have, tend to focus on how home environments influence childhood obesity or diet [10–12]. Given the potentially powerful role that home environments play in shaping dietary behaviors, it is important to identify which dimensions are most influential as potential intervention targets.

Food inventories, or the foods available in the home, are the most commonly studied feature of the home food environment. Several studies have documented associations between home food inventories and a range of diet-related outcomes [5, 13–16]. While most of these studies have focused on children [3, 12, 13, 17], some included parents [14, 18] and a few have focused on adults [5, 15, 16, 19]. Placement of food within the home, or accessibility, is another potentially important dimension of the home food environment. Several studies have shown that accessibility of fruits and vegetables in the home is associated with fruit and vegetable intake in children [10, 12, 13, 20]. Studies of home food accessibility are less common in adults, and report mixed findings [10, 19].

Quality of the food available in the home is dependent on food acquisition behaviors, such as grocery shopping and serving restaurant food for family meals. Grocery shopping behaviors have received a considerable amount of attention in the literature with increased attention on how the food environment influences dietary behavior [21–25], and shopping frequency is correlated with fruit and vegetable intake in several studies [21, 26, 27], but not with obesity in another [25]. Many studies that focus on the role of the home in childhood obesity examine parental practices related to food, such as eating meals in the living/TV area, taking second helpings, eating while watching TV [10], fast food and full service-restaurant food for family meals [28], and food preparation [29, 30]. These same practices are less commonly examined in adults.

At present, there is no consensus and limited research on which features of the home food environment are most influential for diet quality and weight-related behaviors of adults. As part of a weight gain prevention intervention, we developed a conceptual model for home food environments [31, 32] and examined its influence on fruit and vegetable intake and percent energy from fat in low-income overweight and obese women [19]. This model categorizes features of the home food environment into home food availability and cues (e.g., fruit and vegetable inventory, salty snacks/sweets inventory, unhealthy drinks, food placement), household food practices (e.g., food shopping, meal preparation non-home food sources, and household TV and eating), and social support and modeling. The current study extends our past work [19, 31, 32] by characterizing the home food environment of a national sample of U.S. adults and identifying associations with diet quality and overweight/obesity.

## Methods

### Study participants

Participants (*n* = 4942) completed a cross-sectional survey during a two-week period in the fall of 2015. They were recruited from an internet panel using quota rather than probability-based sampling to match U.S. demographics on race/ethnicity, gender, age, income and geographic region. The panel, constructed and maintained by Lightspeed GMI (www.lightspeedresearch.com) is recruited from multiple sources (e.g., social media, banner ads, opt-in email, networks). We obtained completed surveys from (39.9%) of 12,396 individuals who consented to participate. Those not satisfying enrollment quotas (*n* = 3811, 30.7%), not completing the survey (*n* = 2994, 24.2%), and failing an in-survey quality control checks (*n* = 649, 5.2%) were excluded. Study participants lived in the U.S., could read English, and were 18 to 75 years of age. The study protocol was approved by the Emory University Institutional Review Board.

### Measures

Self-reported height and weight were used to calculate BMI, which was then dichotomized into underweight/normal (BMI < 25 kg/m^2^) and overweight/obese (BMI ≥ 25 kg/m^2^) for analyses [33]. Intake (frequency and amount) for nine categories of fruit or vegetables were used to calculate total cups of fruit and vegetables consumed per day using a validated measure [34] and then dichotomized based on the Dietary Guidelines for Americans 2015–2020 [35]. Those consuming 4.5 cups or more of fruit and vegetables per day were classified as meeting the guidelines. The validated National Cancer Institute Quick Food Scan was used to assess percent energy from fat [36].

We assessed eleven dimensions of the home food environment, three related to *household food inventories*. We asked about the availability of 16 fruits, 21 vegetables, 23 snacks and sweets, and eight beverages in the home during the past 7 days [10, 15, 37, 38]. For fruits and vegetables, respondents were able to add additional fruits and vegetables in an open-ended field. For analyses reported here, we included three high fat/sugar beverages and eight high fat/sugar snacks/sweets. We also asked about frequency of *purchasing fruits and vegetables* in the past month, with response options of less than once per week, once per week, and more than once per week. Responses were dichotomized to less than once per week and one or more times per week [31].

*Food placement* measures consisted of three items that asked how often fruits, vegetables, and high calorie snack foods are kept in a place where they are easily seen and reached [10] using a 5-point scale from never to almost always. An overall food placement score was created by reverse coding the snack item and combining it with the fruit and vegetable placement items.

A total of 15 items were used to assess frequency of healthy and less healthy *meal preparation,* with a Cronbach’s alpha of .80 [31, 39]. Items asked about food preparation methods (e.g., trim fat/skin from meat/chicken before cooking), specific cooking methods (e.g., use of a steamer, grill, added fats), and types of food served (e.g., fried foods, reduced fat/calorie milk, dressing, and frozen desserts, and fruits or vegetables). *Portion control practices* were assessed through three items (i.e., use of smaller plates, limiting portions, “family-style” meals) and combined to form an overall score. Response options were 1 = never to 5 = almost always. Overall scores were created for each, with items reverse coded as appropriate.

*Family (or household) meals from restaurants* was assessed by asking how often in a typical week family meals were obtained from fast-food restaurants, full-service/sit-down restaurants, or takeout/delivery. For respondents who lived alone, items from a parallel question about their own meals from each of these sources was used [40]. An overall score was created by summing the days per week each type of restaurant was used, and then creating a daily average.

Using measures adapted from Spurrier and colleagues [11], all participants were asked two questions about how often they personally *ate meals and snacks while watching TV*. Participants who reported living with others answered two similar questions about household meals and snacks. The former was used for respondents who lived alone and the latter for those who had more than one person in the household. These questions used a five-point scale, with responses ranging from never to almost always. One question determined whether any televisions were in the room where main meals were eaten [11]. The final home environment measure asked whether they owned a scale for monitoring body weight.

#### Demographics

Respondents were also asked about their age, sex, race/ethnicity, educational attainment, annual household income, marital status, household size and composition, employment status [33, 41], and to classify the area around their home as rural, small town, suburban, or urban.

### Data analysis

All analyses were conducted using SAS (SAS version 9.4, SAS Institute Inc., Cary, NC., USA). Descriptive statistics were used to examine demographic, home food environment, BMI and diet-related variables. Independent t-tests, Chi-square tests of independence, and Pearson correlations were used to explore bivariate relationships between features of the home food environment, overweight/obesity and dietary intake. Multivariable linear and logistic regression models were used to assess relationships between features of the home food environment and overweight/obesity (BMI ≥ 25), meeting of fruit and vegetable guidelines, and percent calories from fat, adjusted for gender, age, race, income, and type of neighborhood.

## Results

### Description of survey respondents

Table 1 describes the survey respondents. The mean age was 44.4 (SD = 15.4) and 48.3% were men. About one-third had children in the home (32.7%) and 23.1% lived alone. Mean household size was 2.6 (SD = 1.58). The majority were White (67.7%), with 12.3% Black and 14.3% Hispanic. Approximately half (50.1%) had college or graduate degrees, 30.7% reported some college or technical school, and 19.2% reported a high school education or less. Close to one-third reported an annual household income over $75,000 (32.5%) and 25.3% reported less than $25,000. A normal BMI was reported most often (37.1%) with 29.5% overweight and 30.2% obese. Only 17.9% consumed 4.5 cups of fruit and vegetables per day, and the mean percent calories from fat was 35.1 (SD = 5.23).

**Table 1:** Description of Survey Respondents (*n* = 4942)

**Characteristic**		
**Age,** mean, SD	44.4	15.4
**Sex,** n, % male	2389	48.3%
**Children < 18 in the home***, n, %*	1617	32.7%
**Live Alone,** n, %	1142	23.1%
**Household size**, mean, SD	2.6	1.58
**Race/ethnicity,** n, %		
White	3335	67.7%
Black	604	12.3%
Hispanic	703	14.3%
Asian	254	5.2%
Other	29	0.6%
**Education,** n, %		
HS diploma, GED, or less	949	19.2%
Some College or Technical School	1519	30.7%
College Degree or More	2474	50.1%
**Employed, n, %**	2786	56.4%
**Annual Household Income,** n, %		
< $25,000	1251	25.3%
$25,000 to $49,999	1191	24.1%
$50,000 to $74,999	893	18.1%
$75,000+	1607	32.5%
**BMI,** n*, %		
15–18.4	155	3.3%
18.5 to < 25	1763	37.1%
25 to 29.9	1402	29.5%
≥ 30	1435	30.2%
**Fruit and Vegetable Intake,** n, %		
Meet fruit and vegetable intake guidelines (4.5 cups/day)	886	17.9%
% calories from fat*, mean, SD	35.1	5.23

N for BMI = 4755 (187 missing), N for Percent calories from fat = 4938 (4 missing)

### Description of the home food environment

Table 2 presents a description of the home food environment, including the most commonly available fruits and vegetables. On average, respondents reported a mean of 5.2 (SD = 3.65) different varieties of fruits in the home, with apples (74.5%), bananas (72.2%) and grapes (48.2%) most common. Respondents reported 9.1 (SD = 4.79) varieties of vegetables in the home, with onions (71.1%), carrots (71.1%) and tomatoes (70.8%) most common. Total varieties of fruits and vegetables in the home averaged 14.3 (SD = 7.71) across respondents. Of the eight salty snacks and sweets assessed, an average of 3.8 (SD = 2.08) kinds were available in the home; 69.9% of households had chocolate/candy/candy bars and 62.1% had cookies/cakes/snack cakes/donuts. Potato chips were available in 61.5% of households. Of the three high-fat/high-sugar beverages assessed, households had a mean of 1.5 kinds (SD = 1.02) available in the home. Over half (61.5%) of households reported whole milk (61.5%) and regular soft drinks (55.4%). The mean food placement score was 2.5 (SD = 0.69), meaning that unhealthy foods were between sometimes and frequently kept out of sight and easy reach, and fruits and vegetables were easy to see and reach.

**Table 2:** Household Food Inventory and Household Food Practices (*n* = 4942)

Feature	N or Mean	Percent or SD
**Total varieties of fruit** (of 17), mean, (SD)	5.2	(3.65)
Top 5, n, %		
*Apples*	3684	74.5
*Bananas*	3569	72.2
*Grapes*	2382	48.2
*Oranges*	2230	45.1
*Strawberries*	1945	39.4
**Total varieties of vegetables** (of 22), mean, (SD)	9.1	(4.79)
Top 5, n, %		
*Onions*	3515	71.1
*Carrots*	3514	71.1
*Tomatoes*	3498	70.8
*Lettuce*	3407	68.9
*Broccoli*	3159	63.9
**Total fruit and vegetables (**of 39), mean, (SD)	14.3	(7.71)
**Total salty snacks/sweets** (of 8), mean, (SD)	3.8	(2.08)
Top 5, n, %		
*Chocolate/candy/candy bars*	3455	69.9
*Cookies/cakes/snack cakes/donuts*	3071	62.1
*Potato chips*	3038	61.5
*Ice cream*	2615	52.9
*Snack crackers*	2381	48.2
**Total less healthy beverages** (of 3), mean, (SD)	1.5	(1.02)
Whole milk	3038	61.5
Regular soft drinks	2737	55.4
Sugar-sweetened beverages, non-soda	1871	37.9
**Food placement scale** (of 3), mean, (SD)	2.5	(0.69)
Fruits, % almost always (easy to see/reach)	2146	43.4
Vegetables, % almost always	1570	31.8
Unhealthy snacks, % almost always^b^	1261	25.5
**Grocery shopping ≥ 1/week**		
For fruit, n, %	3895	78.8
For vegetables, n, %	3773	76.4
**Meal preparation scale**^a^, mean, (SD)	1.9	(0.66)
Buy skinless chicken or remove skin	2.3	(1.37)
Buy lean meat or trim fat	2.2	(1.32)
Use a non-stick pan and no grease	2.2	(1.22)
Serve fruit or vegetables for snacks	2.1	(1.15)
Use spices on vegetables instead of oil, butter or fat	2.1	(1.27)
Broil or bake meat or fish	2.0	(1.24)
Serve fruit for dessert	1.9	(1.22)
Cook with a grill	1.6	(1.21)
Serve 1% or skim milk	1.6	(1.53)
Use low calorie or diet salad dressings	1.4	(1.34)
Use low-fat or non-fat mayonnaise	1.2	(1.34)
Serve non-fat ice cream, frozen yogurt or sherbet	1.1	(1.24)
Cook with a steamer	1.1	(1.22)
Serve fried fish or fried chicken^b^	1.4	(1.18)
Serve fried vegetables like okra or FF^b^	1.2	(1.17)
**Portion control score**^a^, mean, (SD)	1.9	(0.73)
Use smaller plates	1.7	(1.24)
Serve smaller amounts of food	1.7	(1.15)
Serve meals family style^b^	1.7	(1.33)
**Restaurant food for family meals, times/day,** mean, SD	0.7	(0.78)
**Family TV and eating**^a^**,** mean, (SD)	2.5	(1.13)
Meals, n, % almost always	1251	25.3
Snacks, n, % almost always	1153	23.3
**Own scale,** n, % yes	3744	75.8
**TV in dining area, n,** % yes	3041	61.5

^a^Range 1–4 with 1 = never to 4 = Almost always. ^b^Reverse coded for mean calculation

The majority of respondents reported shopping for both fruits and vegetables at least once a week, 78.8% for fruit and 76.4% for vegetables. The mean meal preparation score was 1.9 (SD = 0.66) which corresponds with sometimes. The most common healthy food preparation behaviors were related to meat purchasing or preparation and using a non-stick pan with no grease. Serving a range of low fat food was less common (e.g., low-calorie salad dressing, low-fat mayonnaise). The portion control score was also 1.9 (SD = 0.73), with little differentiation across specific practices.

Various types of restaurants, including fast food, sit-down and take out/delivery, were used for family/household meals a mean of 0.7 times per day (SD = 0.78). Family meals and snacks with the TV on occurred between sometimes and frequently on average, with 25.3% percent stating their household almost always ate meals in front of the TV and 23.3% stated their household almost always ate snacks in front of the TV.

The majority owned a scale (75.8%) and had a TV in the room where main meals were eaten (61.5%).

### Bivariate associations between features of the home food environment, diet quality, and obesity/overweight

All features of the home food environment were significantly associated with meeting national fruit and vegetable guidelines in bivariate analyses (Table 3), including diversity of the fruit and vegetable inventory (20.6 for met versus 13.0 for unmet), food placement (2.7 for met versus 2.4 for unmet), shopping for fruit at least once per week (90.9% versus 76.2%), meal preparation (2.3 versus 1.8), portion control (2.1 versus 1.9), and owning a scale (81.4% versus 74.5%). Salty snacks/sweets (4.5 for met versus 3.6 for unmet), unhealthy beverages (1.8 for met versus 1.1 for unmet), family eating in front of the TV (2.6 versus 2.4), a TV in the dining area (70.5% versus 59.6%), and family meals from restaurants (1.2 versus 0.6) were also associated with meeting of guidelines.

**Table 3:** Bivariate Associations between Features of the Home Food Environment, Diet Quality and BMI

Feature	Fruit & Vegetable Guidelines (***n*** = 4942)					Percent Calories from Fat (***n*** = 4938)			BMI Classification (***n*** = 4755)				
	Met		Unmet						Under/Normal Weight (BMI < 25)		Overweight/Obese (BMI ≥ 25)		
	**Mean**	**(SD)**	**Mean**	**(SD)**	***p*-value**	**r**		***p*-value**	**Mean**	**(SD)**	**Mean**	**(SD)**	***p*-value**
Fruit & vegetable inventory (of 39)	20.6	(7.99)	13.0	(6.94)	<.0001	0.15		<.0001	14.9	(7.89)	13.5	(7.29)	<.0001
Salty snacks/sweets inventory (of 8)	4.5	(2.26)	3.6	(2.00)	<.0001	0.25		<.0001	3.8	(2.08)	3.7	(2.06)	0.11
Less healthy beverages inventory (of 3)	1.8	(1.07)	1.1	(1.01)	<.0001	0.24		<.0001	1.5	1.03	1.5	(1.02)	0.40
Food placement	2.7	(0.62)	2.4	(0.70)	<.0001	−0.14		<.0001	2.5	0.69	2.5	(0.70)	0.048
Meal preparation	2.3	(0.63)	1.8	(0.63)	<.0001	0.05		0.0004	1.9	(0.66)	1.8	(0.64)	<.0001
Portion control	2.1	(0.66)	1.9	(0.74)	<.0001	0.03		0.032	1.9	(0.72)	1.9	(0.74)	0.65
Restaurant food for family meals	1.2	(1.01)	0.6	(0.67)	<.0001	0.41		<.0001	0.7	(0.80)	0.65	(0.71)	0.0002
Family TV and eating	2.6	(1.07)	2.4	(1.15)	0.002	0.18		<.0001	2.3	(1.14)	2.5	(1.13)	<.0001
	**N**	**%**	**N**	**%**	***p*-value**	**Mean** **BMI**	**(SD)**	***p*-value**	**N**	**%**	**N**	**%**	***p*-value**
Shopping for fruit, ≥1/week													
Yes	805	90.9	3090	76.2	<.0001	35.0	(5.34)	0.035	1562	81.4	2185	77.0	0.0003
No	81	9.1	966	23.8		35.4	(4.83)		356	18.6	652	23.0	
Own scale													
Yes	721	81.4	3023	74.5	<.0001	35.2	(5.45)	0.18	1464	76.3	2141	75.5	0.50
No	165	18.6	1033	25.5		34.9	(4.51)		454	23.7	696	24.5	
TV in dining area													
Yes	625	70.5	2416	59.6	<.0001	35.7	(5.60)	0.18	1133	59.1	1758	62.0	0.04
No	261	29.5	1640	40.4		34.1	(4.42)		788	40.9	1079	38.0	

Note: independent t-tests, chi-square tests of independence, and ANOVA

Nine of the home food environment features were associated with percent calories from fat, although correlations were generally modest as shown in Table 3. The highest correlations were for family meals from restaurants (*r* = .41), salty snacks/sweets (*r* = .25), unhealthy beverages (*r* = .24), and eating with the TV on (*r* = .18). Meal preparation, portion control were significant, but not meaningfully correlated. Food placement was negatively correlated (*r* = −.14), fruit and vegetable inventory was positively associated with percent calories from fat (*r* = .15). Shopping frequency for fruit was associated with fat intake, but the difference was too small to be meaningful. TV in the dining area and owning a scale were not associated with percent calories from fat.

Fewer features of the home food environment were associated with overweight/obesity, with salty snacks/sweets inventory, less healthy beverages inventory, portion control practices, and owning a scale not related. Features associated with overweight/obesity included fruit and vegetable inventory (14.9 for under/normal weight versus 13.5 for overweight/obese), shopping for fruit (81.4% versus 77.0%), meal preparation (1.9 versus 1.8), watching TV while eating (2.3 versus 2.5), TV in the dining area (59.1% versus 62.0%), and household meals from restaurant food (0.73 for under/normal weight versus 0.65 for overweight/obese). The association with food placement was also significant but too small to be meaningful.

### Multivariable analyses

Table 4 presents regression models that examine which dimensions of the home food environment were associated with meeting national guidelines for fruit and vegetable intake, percent calories from fat, and overweight/obesity. While controlling for demographic variables and type of neighborhood based on level of rurality, seven of 11 features of the home food environment were associated with meeting fruit and vegetable intake guidelines. The strongest associations were restaurant food for household meals (OR = 1.73; CI 1.54, 1.94), food placement (OR = 1.49, CI 1.28, 1.74), meal preparation (OR = 1.48, CI 1.25, 1.76), and frequency of shopping for fruit (OR = 1.52, CI 1.14, 2.01). Having more types of fruit and vegetables in the home (OR = 1.09, CI 1.08, 1.11) was also associated with meeting the guidelines, as were having a TV in the dining area (OR = 1.36, CI 1.11, 1.67), and having more salty/sweet snacks in the home (OR = 1.06, 1.01, 1.12).

**Table 4:** Regression Models for Home Food Environments, Dietary Intake and Overweight/Obesity

Feature of the Home Food Environment	Logistic Regression Model Met Fruit & Vegetable Guidelines (***n*** = 4921)		Multiple Regression Model for Percent Calories from Fat (***n*** = 4917)			Logistic Regression Model for Overweight/Obesity (***n*** = 4735)	
	OR	(95% CI)	b	SE	***p***-value	OR	(95% CI)
Fruit & vegetable inventory	**1.09**	**(1.08, 1.11)**	**0.06**	**0.01**	**<.0001**	**0.98**	**(0.97, 0.99)**
Salty snacks/sweets inventory	**1.06**	**(1.01, 1.12)**	**0.23**	**0.04**	**<.0001**	1.00	(0.97, 1.04)
Less healthy beverages inventory	0.95	(0.86, 1.04)	**0.51**	**0.07**	**<.0001**	1.04	(0.97, 1.12)
Food placement	**1.49**	**(1.28, 1.74)**	**−0.37**	**0.11**	**0.0008**	1.04	(0.94, 1.15)
Meal preparation	**1.48**	**(1.25, 1.76)**	**−0.45**	**0.13**	**0.0006**	1.02	(0.90, 1.16)
Portion control	1.04	(0.91, 1.18)	0.08	0.10	0.4024	1.03	(0.94, 1.13)
Restaurant food for family meals	**1.73**	**(1.54, 1.94)**	**2.17**	**0.10**	**<.0001**	0.97	(0.88, 1.06)
Family TV and eating	0.97	(0.89, 1.06)	**0.32**	**0.07**	**<.0001**	**1.14**	**(1.07, 1.21)**
Grocery shopping for fruit	**1.52**	**(1.14, 2.01)**	**−0.50**	**0.18**	**0.0047**	0.95	(0.80, 1.13)
Own scale	1.04	(0.84, 1.29)	−0.01	0.16	0.9327	0.98	(0.84, 1.13)
TV in dining area	**1.36**	**(1.11, 1.67)**	0.29	0.15	0.0601	0.99	(0.86, 1.14)

All models controlled for gender, race, age, income and neighborhood rurality/urbanicity

Eight of 11 features of the home food environment were associated with percent energy from fat in the multivariable analysis. The strongest association was between restaurant food for household meals (i.e., fast food, sit down restaurants, or take-out/delivery) and percent calories from fat (b = 2.17, SE =0.10, *p* < .0001). Other associations were salty snacks and sweets (b = 0.23, SE = 0.04, *p* < .0001), unhealthy beverages (b = 0.51, SE = 0.07, *p* < .0001), food placement (b = − 0.37, SE = 0.11, *p* = 0.0008), meal preparation (b = − 0.45, SE = 0.13, *p* = 0.0006), family eating with the TV on (b = 0.32, SE = 0.07, *p* < .0001), and frequency of shopping for fruit (b = − 0.50, SE = 0.18, *p* = 0.005). Fruit and vegetable inventory was also associated with percent calories from fat (b = 0.06, SE = 0.01, *p* < .0001), while portion control, having a TV in the dining room, and owning a scale were not.

Just two of the home food environment features were associated directly with overweight/obesity and these associations were small. More frequent household meals and snacks while watching TV was associated with increased odds of overweight/obesity (OR = 1.14, CI 1.07, 1.21), and an increased fruit and vegetable inventory was associated with lower odds of overweight/obesity (OR = 0.98, CI 0.97, 0.99).

## Discussion

This paper characterizes multiple dimensions of the home food environments of U. S adults using a national sample. Prior research on home food environments generally examines sub-populations, often using baseline data from intervention trials, families with children, or a limited set of home food environment features. To our knowledge, this is the first study to examine multiple features of the home food environment using a national sample. Other national studies have used NHANES data to examine one or two dimensions [6, 42, 43]. Using NHANES data from 2007 to 2010, for example, Chai et al. [6] found that 85.4% of adults had fruits available in their home always or most of the time. Our study also showed relatively high fruit availability, with over 70% of households reporting apples and/or bananas in the home during the past 7 days. Chai et al. reported that 62.5% reported salty snacks, this is comparable to our finding that over 60% of households had regular potato chips in their homes. Additionally, Chai reported that 56.3% had sugary drinks available in the home all or most of the time. We found that over 50% had regular soft drinks in their home and over 35% had non-soda sugar sweetened beverages.

It is more difficult to compare findings on other dimensions of the home food environment across studies given measurement differences and a focus on specific demographic groups or just one or two geographic areas [4, 7, 10, 18, 44]. Because we used many measures from our earlier study of home food environments among overweight and obese women in rural Georgia [19, 31], we can make some comparisons. In that study, there was an average of 14.0 varieties of fruits and vegetables available in the home compared to 14.3 in the current study. Other findings were also similar, with the exception of a higher portion control (called meal serving in the earlier study) score in the study of overweight/obese women than in the current study. This may be due to actual behavioral differences related to weight management strategies or social desirability.

To our knowledge, the current study is also the first to explore associations of multiple dimensions of the home food environment with important public health outcomes, including fruit and vegetable intake, percent calories from fat, and overweight/obesity in a national sample. We examined eleven dimensions of the home food environment and found that a majority were related to diet quality (e.g., meeting of fruit and vegetable guidelines and percent calories from fat) and two were associated with overweight and obesity in multivariable models. Taken together, the relationships observed in this study begin to show a pattern that may be instructive for future interventions. Fewer home food environment variables were associated with overweight/obesity in either bivariate or multivariate analyses, perhaps reflecting the complex relationship between weight, food, and myriad other factors, plus the more proximal nature of diet quality relative to weight.

Our finding that the varieties of fruits and vegetables present in the home was associated with an increased likelihood of meeting national fruit and vegetable guidelines is consistent with earlier studies, many of which have shown associations between fruit and vegetable inventory and fruit and vegetable intake in children [13, 14, 17] and also adults [45]. Consistent with at least one other study [44], we also found that fruit and vegetable inventory was associated with a decreased likelihood of overweight/obesity. Surprisingly, we found that fruit and vegetable inventory was associated with increased percent energy from fat.

Other components of the home food inventory were also associated with dietary behavior. Both salty snacks and sweets, and unhealthy beverage inventories, were associated with higher levels of percent calories from fat. Somewhat surprisingly, a higher inventory of salty snacks and sweets was associated with increased likelihood of meeting fruit and vegetable intake guidelines. Few other studies have documented the constellation of snacks in the home and dietary outcomes. Among those that have, positive associations were noted between home availability of high fat snacks with fat intake [5, 15] and overweight [44].

We found that food placement was associated with decreased percent calories from fat and increased likelihood of meeting national fruit and vegetable guidelines. In our earlier study on overweight and obese women, we did not find any associations between food placement and fruit and vegetable intake or percent calories from fat [19]. Several studies had documented that increased accessibility was associated with fruit and vegetable intake in children [10, 12, 13, 20].

We also found that healthier meal preparation was associated with both increased likelihood of meeting fruit and vegetable intake guidelines and lower percent energy from fat. While few studies have examined meal preparation as a dimension of home food environments, several studies have examined whether food preparation methods are associated with diet and weight, yielding mixed results [19, 29, 30]. Trude et al. [30] reported that household food preparation was not associated with fruit and vegetable intake among adolescents, unless the adolescents were directly involved in the preparation. A study by Kramer and colleagues reported lower BMI among African American youth whose households used healthier food preparation methods [29]. Our earlier study found that healthy food preparation was associated with lower percent calories from fat [19].

Our portion control measure was not associated with any of the outcomes we examined in multivariable analyses. Despite studies that document how environmental factors such as plate size can influence amount of food consumed in laboratory settings [46, 47], to our knowledge, very few studies have examined whether behaviors to limit family portion sizes are associated with dietary behaviors or weight.

Shopping for fruit was associated with fruit and vegetable intake. Frequency of shopping has been shown to be associated with fruit and vegetable intake in a couple of studies [19, 21, 26]. We found that increased frequency of restaurant food for family meals was associated with increased percent energy from fat, and surprisingly, increased meeting of fruit and vegetable consumption, even when controlling for income. Boutelle et al. found that purchase of fast food for family meals was associated with lower vegetable intake and higher BMI among parents of adolescents [28]. Frequency of restaurant eating in general (i.e., not limited to family meals) has been examined in a larger number of studies, with findings that show frequency of away from home meals (i.e., fast food and full-service restaurant food and beverage consumption) is associated with increased energy intake and BMI [48, 49].

Family meals and snacks with the TV on were associated with both percent intake from fat and overweight/obesity in our study. Past studies have shown that frequency of TV on during dinner was associated with lower F & V consumption in adults [50]; and that TV watching while eating was associated with percent energy from fat [51]. A TV in the dining room, however, was associated only with fruit and vegetable guidelines being met in our study. Other studies have shown that TV watching is associated with obesity [52]. Gorin found that more TVs in the household were associated with overweight and obesity [44]. Lastly, owning a scale was not associated with overweight/obesity or either of the diet quality outcomes in our study.

This study has a few important limitations. First, because the data are from an online sample, those without internet services are likely under-represented, as are those without a college degree. Although the sample was designed to match the U. S population on a few key characteristics, it is not probability-based, therefore inferences cannot be made to the full population. Second, the data are self-reported, and thus, susceptible to social desirability bias. Third, the data are from 2015 and do not reflect the possibly major changes made to home food environments as a result of the COVID-19 pandemic. We acknowledge that the home food environment is situated within a larger community food environment and that additional research is needed on the ways in which the home environment may mediate the association between the larger food environment and dietary outcomes. Nevertheless, this study makes an important contribution by providing one of the most comprehensive examinations of the home food environment published to date.

## Conclusion

In this cross-sectional study, numerous dimensions of the home food environment, including food and beverage inventories, food placement, meal preparation, frequency of shopping, use of restaurant meals, and TV and eating practices, were associated with diet quality. A more diverse fruit and vegetable inventory was associated with lower odds of overweight/obesity, and more frequent family eating while watching TV was associated with increased odds of overweight/obesity. These findings have implications for how to improve diet quality. Although more intervention research is needed, targeting dimensions of the home identified in this study with motivational interviewing, goal setting or social marketing strategies may be a promising approach consistent with our understanding that environments can constrain and encourage health behaviors, including healthy eating. Changing the larger food environment in which home environments are situated may be an important complementary strategy. A deeper understanding of which dimensions can drive improvements in diet quality and healthy weight maintenance may strengthen current approaches to nutrition and obesity interventions.

## Data Availability

The dataset for the current study is available from the corresponding author on reasonable request.

## Abbreviations

BMI: Body mass index
OR: Odds ratio
